# A novel signature constructed by differential genes of muscle-invasive and non-muscle-invasive bladder cancer for the prediction of prognosis in bladder cancer

**DOI:** 10.3389/fimmu.2023.1187286

**Published:** 2023-08-24

**Authors:** Weizhuo Wang, Xi Zhang, Silin Jiang, Peng Xu, Kang Chen, Kai Li, Fei Wang, Xiang Le, Ke Zhang

**Affiliations:** ^1^ Department of Urology, The Affiliated Suzhou Hospital of Nanjing Medical University, Suzhou Municipal Hospital, Gusu School, Nanjing Medical University, Suzhou, China; ^2^ Department of Urology, Department of Urology, The First Affiliated Hospital of Nanjing Medical University, Nanjing, China; ^3^ Department of Urology, Department of Urology, The Second Affiliated Hospital of Nanjing Medical University, Nanjing, China; ^4^ Department of Urology, North China University of Science and Technology, Tangshan, China

**Keywords:** bladder cancer, muscle-invasive, prognostic signature, immune microenvironment, biomarker

## Abstract

**Background:**

Bladder cancer (BCa) is a malignant tumor that usually forms cancer cells in the inner lining of the bladder. Hundreds of thousands of people worldwide have BCa diagnosed each year. The purpose of this study was to construct a prognostic model by differential expression of genes between muscular and non-muscular invasive BCa, and to investigate the prognosis of BCa patients.

**Methods:**

The data of BCa patients was sourced from the GEO and TCGA database. Single-cell sequencing data was obtained from three patients in the GSE135337 database, and microarray data for verification was obtained from GSE32894. Univariate, Lasso and multivariate cox regression analyses were performed to construct the prognostic model. The prognostic features, immune features and drug sensitivity of the model were further evaluated. Single-cell data and microarray data were used to validate the differential expression of model genes between muscle-invasive and non-muscle-invasive BCa. The invasion and migration of BCa cells were evaluated using the transwell assay and wound-healing assay. The cell proliferation capacity was simultaneously evaluated using Colony formation experiments. The protein expression of the specific gene was detected by western blot analysis.

**Results:**

We identified 183 differentially expressed muscle-invasive-related differential genes (MIRDGs), among which four were selected to establish a prognostic model. Based on our signature, patients in different groups displayed varying levels of immune infiltration and immunotherapy profiles. Single-cell sequencing data and microarray data confirmed that four invasion-related genes were expressed at higher levels in muscle-invasive BCa. Given the critical role of S100A9 in the progression of BCa, we performed further analysis. The results showed that protein expression of S100A9 was high in muscle-invasive BCa, and S100A9 knockdown could inhibit the proliferation, migration and invasion of BCa.

**Conclusion:**

These findings demonstrated that the prognostic model for BCa patients was reasonably accurate and valid, and it may prove to be of considerable value for the treatment and prognosis of BCa patients in the future. S100A9 may become a better prognostic marker and potential therapeutic target to further guide clinical treatment decisions.

## 1 Introduction

Bladder cancer (BCa) is a type of malignant tumor that typically forms cancer cells in the inner layer of the bladder. Hundreds of thousands of people are diagnosed with BCa worldwide every year, and its incidence is increasing year by year (1). BCa is common in older people, while in developing countries, it is often related to work environments and environmental pollution. According to statistics, men are more likely than women to develop cancer, and factors such as smoking, long-term exposure to chemicals, and chronic cystitis are also related to cancer (2).

Regarding the treatment of BCa, existing researches show that whether infiltration of the muscle layer is present is a key factor in determining a patient’s treatment plan. Based on this, BCa patients can be divided into non-muscle-invasive and muscle-invasive types. Among them, 60%-70% of non-muscle-invasive types are confined to the bladder mucosa (Ta stage), 20%-30% show subepithelial connective tissue infiltration (T1 stage), and about 10% show *in situ* carcinoma. The main treatment for non-muscle-invasive BCa is transurethral resection of bladder tumor (TURBT), followed by immediate injection of bacillus Calmette-Guerin (BCG) vaccine or intravesical chemotherapy. The decision to administer BCG and/or chemotherapy is based on the risk of cancer progression or recurrence. Muscle-invasive BCa invades the muscle layer, including invasion of the muscle layer (T2 stage), invasion of surrounding tissue (T3 stage), and invasion of any surrounding organ such as the prostate, seminal vesicles, uterus, vagina, pelvic wall, abdominal wall (T4 stage), etc. Given the invasiveness of muscle-invasive BCa, timely diagnosis and treatment are crucial (3). Existing treatments strongly recommend radical cystectomy with bilateral pelvic lymph node dissection and platinum-based neoadjuvant chemotherapy for all resectable non-metastatic muscle-invasive BCa patients (4).

The prognosis and treatment of muscle-invasive and non-muscle-invasive BCa are significantly different, and the likelihood of recurrence and poor prognosis is higher in muscle-invasive BCa (5). Most non-invasive BCa only require bladder resection and instillation therapy, while muscle-invasive BCa requires bladder removal and surrounding cleaning, and even bladder reconstruction, but this method has no evidence of improving long-term outcomes and has a significant impact on the patient’s life (6). Non-muscle-invasive progresses to muscle-invasive BCa in approximately 10% to 20% of cases, requiring continuous follow-up and subsequent treatment (7).Additionally, the treatment of BCa often involves a significant amount of follow-up and subsequent treatment, which can place a heavy financial burden on patients (8). For example, the recommended Bacillus Calmette-Guérin (BCG) attention treatment for non-invasive BCa patients in current guidelines costs approximately $100,000 in the first year alone (9). For non-invasive BCa patients, an effective target that can identify their prognosis is needed to roughly determine their possible progression and adjust the treatment plan accordingly. In clinical practice, sometimes patients experience repeated recurrence despite transurethral resection and BCG treatment (10). In such cases, more aggressive treatment methods, such as partial bladder resection or bladder removal surgery, should be considered to prevent disease progression. Pathological grading, recurrence frequency, and growth range of the tumor are used as means of judging the situation. With the rapid development of genomic technologies, the cost of sequencing is also decreasing. The cost of single-sample sequencing is now under $100,especially for clinical exome sequencing (CES), and if a unified platform is used for sequencing, the cost is likely to decrease further (11). This is a small fraction of the long-term follow-up and treatment costs for BCa patients. In addition, standardized sequencing at scale will standardize the various gene expression values that affect patient prognosis.

In the process of BCa, various genes such as TFPI-2 and GATA3 have been revealed to cause a change in the invasive ability of BCa (12, 13). For the specific staging diagnosis of BCa, clinical identification often relies on samples obtained through transurethral resection of bladder tumor (TURBT) (3).Convenient sample acquisition provides good convenience for gene sequencing that can be performed. Although recent studies have shown that detecting urinary methylation levels contributes to the diagnosis and prognostic prediction of BCa, the current gold standard diagnostic method remains TURBT. In existing research, certain biomarkers have been identified for their value in the progression and prognosis of BCa. For example, BUB1 has been found to predict the prognosis of non-muscle-invasive BCa (14), while STAG2 has demonstrated independent prognostic value in low-grade non-muscle-invasive BCa (15). Regarding the prediction of BCa recurrence, BCL-2 Family, p63 have been considered to have some clinical value (16). In existing research, several genes have been identified to have an impact on the prognosis of bladder cancer, such as SERPINE2, SNCAIP, S100A9, and others (17–19). Particularly, S100A9 has been shown to be highly expressed in bladder cancer tissues in large-scale clinical studies (20), and its expression levels are also elevated in the urine of patients (21). However, there is limited research on its role in muscle-invasive and non-muscle-invasive bladder cancer. Existing studies often rely on databases, and there is a lack of comparative analysis regarding its influence on bladder cancer recurrence and progression. Therefore, further exploration and standardized guidelines are still needed before these genes can be clinically applied.

## 2 Method

### 2.1 Data source

The consolidated transcriptome expression matrix and clinical data of BCa were obtained from the Cancer Genome Atlas (TCGA) and Gene Expression Omnibus (GEO) databases. GSE13507 dataset was used as the training cohort for building the model, while the TCGA-BLCA dataset was used as the testing cohort. The single-cell sequencing data used to validate the differential gene expression of the model came from the GSM4006647, GSM400644, and GSM4006645 in the GSE135337 dataset. The microarray data used to validate the differential gene expression came from the GSE32894 dataset.

### 2.2 Differential genes in the muscle-invasive and non-muscle-invasive bladder cancer

After downloading the gene expression data from GSE13507, the data was organized and analyzed for differential expression using the limma package in R language (22). MIRDGs were selected based on criteria such as |Log2FC|>1 and adj.P.Val <0.05, and visualized in a volcano plot using the ggplot2 package. Enrichment analysis was performed on the resulting MIRDGs (23), and a heatmap was generated to display the results. Additionally, a protein-protein interaction network was constructed for the MIRDGs (24).

### 2.3 Construction of bladder cancer related prognostic model

We employed statistical analyses to investigate the impact of genes on patient prognosis. Specifically, we first used univariate cox regression analysis on training cohort GSE13507 to identify prognostic genes, and presented the results using a heatmap. Subsequently, we employed the least absolute shrinkage and selection operator (LASSO) Cox regression and multivariate Cox regression analysis to construct the bladder cancer related prognostic model. The patients were then divided into either the low risk group or the high risk group based on the median riskscore. TCGA-BLCA was used as the testing cohort to verify the accuracy of the prognostic model. The prognostic accuracy of the riskscore was evaluated using kaplan-meier (KM) analysis, the area under the curve (AUC) of the receiver operating characteristic (ROC) curve, as well as univariate and multivariate independent prognostic analysis.Then we construct a nomogram based on the training cohort (GSE13507).

### 2.4 Single-cell data and microarray data validation data exist for differential expression of the genes identified by the model

We integrated the pTa (GSM4006644), pT2 (GSM4006647), and pT1 (GSM4006645) samples in order to compare the expression patterns of model genes in the single-cell data. Microarray data from the GSE32894 dataset were further used to validate the expression differences.

### 2.5 Immune infiltration and immunotherapy analysis

Single-sample gene set enrichment analysis (ssGSEA) was performed using the “GSVA” package to calculate enrichment scores for different immune cell types and immunologic functions using immune-related gene sets (25). The immunosuppressive checkpoints were sourced from relevant literature and the website (https://www.immport.org/home, **Supplementary Table 9**) (26). Tumor microenvironment (TME) may affect the occurrence and development of cancer, so we employed the ESTIMATE algorithm to evaluate the TME score (ImmuneSocre, StromalScore, and tumor purity) of BCa samples. The Gene expression profiling data and clinical results of 348 BCa immunotherapy patients were obtained from in IMvigor210 cohort. The results of anti PD-L1 immunotherapy responses were divided into complete response (CR), partial response (PR), stable disease (SD), and disease progression (PD).

### 2.6 Western blot assay

The tested proteins are derived from 12 postoperative pathological tissue samples obtained from 8 patients. Among these samples, there are four from patients with muscle-invasive BCa and four from patients with non-muscle-invasive BCa. Additionally, four samples of normal tissue were obtained from adjacent tissue to the cancerous area. WB assay was performed after the detection of protein concentration. 20 μg of samples were separated on a 10% SDS-PAGE gel, then transferred to a PVDF membrane and blocked for 1 hour at room temperature. The membranes were incubated with primary antibodies (S100A9 concentration, 0.5 µg/mL; β-actin dilution rate, 1:500; Abmart) at 4°C overnight. The next day, the membranes were incubated with the secondary antibody (Abmart; dilution rate, 1:2000) at 24°C for 1 h. Signals of targeted proteins were detected using an enhanced chemiluminescence detection system.

### 2.7 Wound-healing assay

Cell migration was assessed by performing a wound healing assay. Briefly, T24 and UMUC3 cells were transfected with S100A9. Approximately 2x10^6 cells were seeded into 6-well plates and cultured for 24 h. Then, a yellow plastic pipette tip was used to create a wound by scraping the cells. Cell migration was monitored under a Nicon Eclipse microscope and photographed at 100×.

### 2.8 Cell proliferation assay and transwell assay

Following the standard procedure, the proliferation ability of the cells was assessed with colony formation assays. T24 and UMUC3 cells (with an incubation density of 2x10^5) were incubated in the upper chambers (Labselect). For the invasion assay, the upper chambers were pre-coated with Matrigel (BD Biosciences). Culture medium without and with 10% FBS was added into the upper and lower chambers, respectively. After 12h, non-migrated cells were wiped out while migrated or invaded CRC cells were fixed, stained and counted using an inverted microscope.

## 3 Results

### 3.1 Identification of the differentially expressed genes

**Figure 1:**
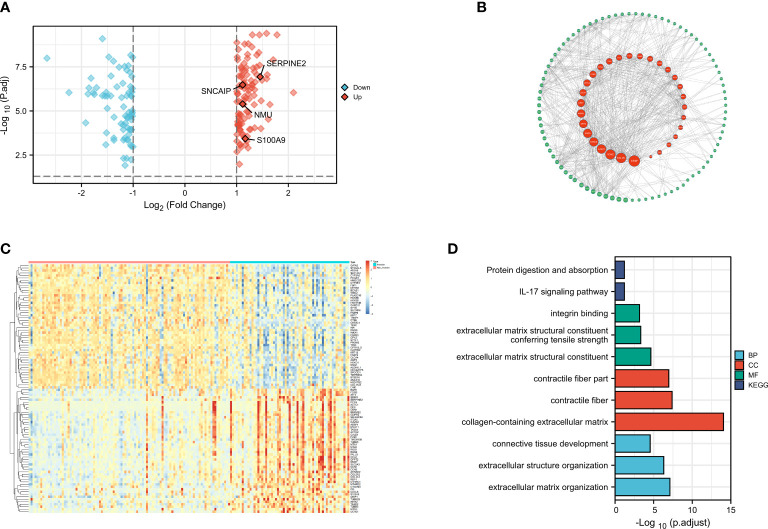
Differential Analysis, Protein Network Construction, GO Analysis, and KEGG Analysis(revised). **(A)** Volcano plot of differential gene muscle-invasive and non-muscle-invasive bladder cancer; **(B)** PPI protein network; **(C)** Heatmap of differential gene muscle-invasive and non-muscle-invasive bladder cancer; **(D)** GO analysis and KEGG analysis.

First, we downloaded the expression matrix and clinical data (**Supplementary Tables 1**, **2**) files of GSE13507 from GEO, including 103 samples of primary non-muscle-invasive BCa and 61 samples of primary muscle-invasive cancer. We then performed differential analysis using the limma package in R language, setting the differential value as |logFC|> 1 and adj.P.Val.< 0.05. A total of 183 DEGs were selected (**Supplementary Table 3**) and we presented them using the volcano plot and heatmap (**Figures 1A, C**).

### 3.2 Enrichment analysis of differentially expressed genes and construction of protein interaction networks

After identifying the DEGs, we performed enrichment analysis on these genes using the org.Hs.eg.db package in R. Our analysis criteria were set at adj.P.Val < 0.1 and q.value < 0.2 (**Supplementary Table 4**). Enrichment analysis of the gene ontology biological process (GO : BP) showed significant enrichment in extracellular matrix organization, extracellular structure organization, connective tissue development, while the cellular component (GO : CC) analysis showed significant enrichment in collagen-containing extracellular matrix, contractile fiber, contractile fiber part. Molecular function (GO : MF) analysis revealed significant enrichment in extracellular matrix structural constituent, extracellular matrix structural constituent conferring tensile strength, integrin binding. Furthermore, KEGG analysis revealed that these DEGs were mainly involved in IL-17 signaling pathway, protein digestion and absorption (**Figure 1B**). These results were consistent with previous studies and suggested that changes in cell adhesion and interaction in the tumor tissue may lead to the invasive changes observed in the tumor as a whole. In addition, a protein-protein interaction (PPI) network analysis was performed using the STRING database (**Supplementary Table 5**), and the PPI network diagram showed that FABP6, ACTC1, and S100A9 had the strongest interactions with other MIRDGs (**Figure 1D**).

### 3.3 Identification of the prognostic features of bladder cancer related prognostic model

**Figure 2:**
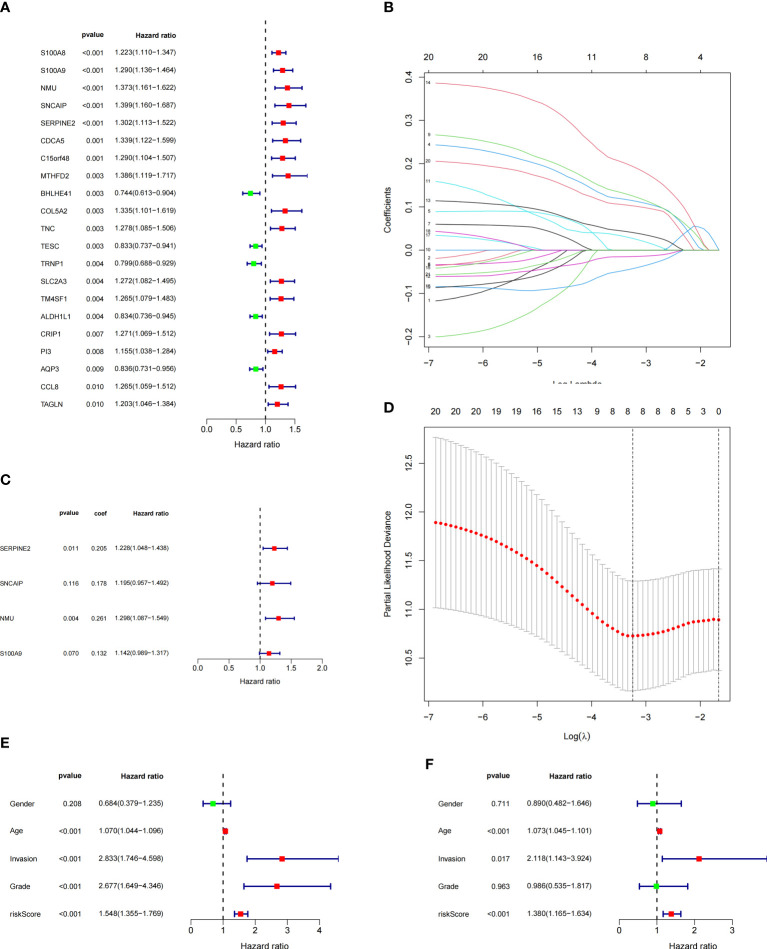
Model Construction and Independent Evaluation(revised). **(A)** Genes identified by univariate cox regression, **(B, D)** lasso regression, **(C)** Model genes identified by multivariate cox regression, **(E)** Univariate independent prognosis analysis, **(F)** Multivariate independent prognosis analysis.

**Figure 3:**
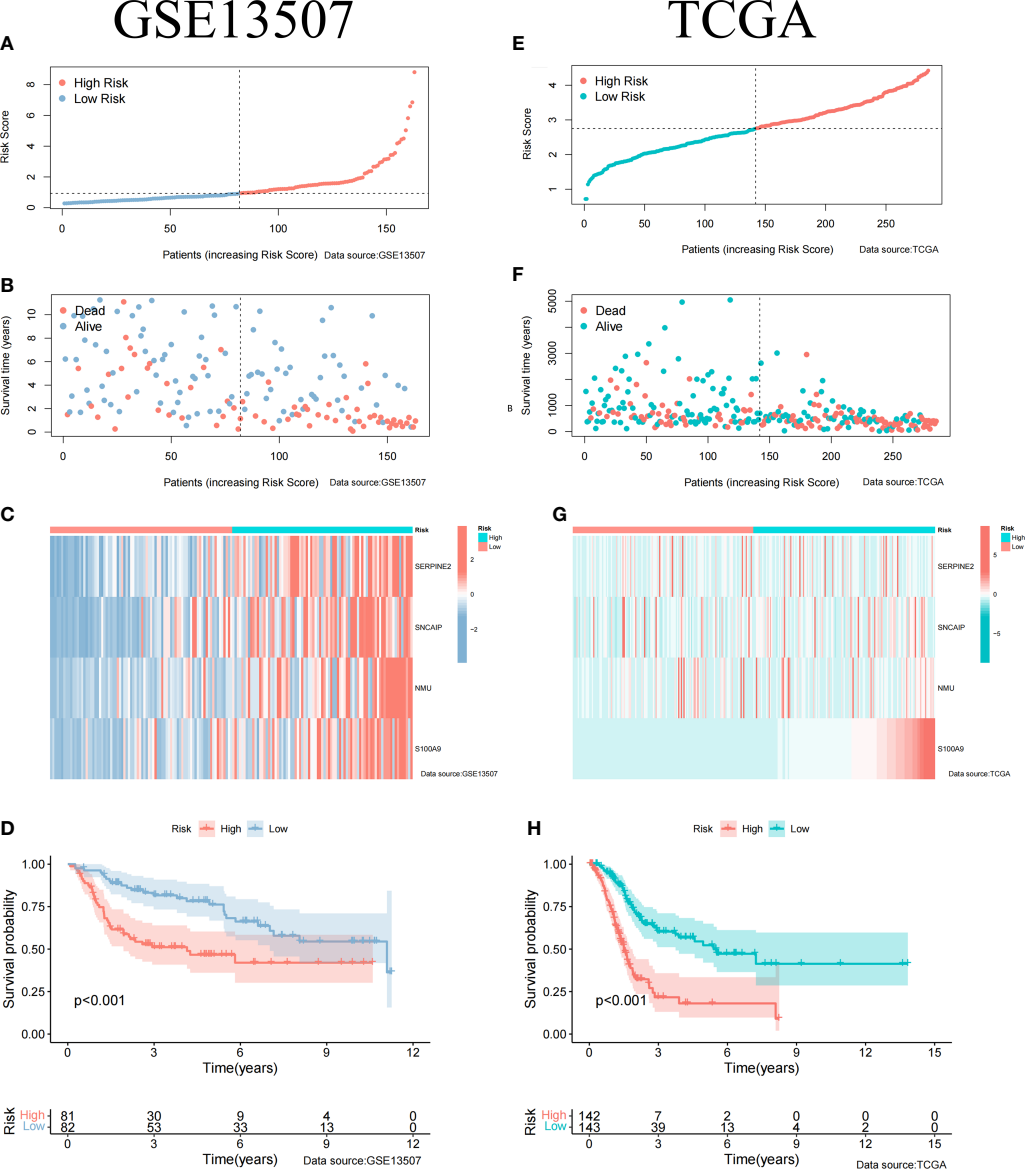
Clinical Relevance of the Model in GSE13507 and TCGA cohort. **(A)** Riskscore information for patients in GSE13507. **(B)** Survival status of patients in GSE13507 with increasing riskscore. **(C)** Gene expression profile of patients in GSE13507 model with increasing riskscore. **(D)** Survival curves of high and low risk groups in GSE13507 patients. **(E)** Riskscore information for patients in TCGA. **(F)** Survival status of patients in TCGA with increasing riskscore. **(G)** Gene expression profile of patients in TCGA model with increasing riskscore. **(H)** Survival curves of high and low risk groups in TCGA patients.

**Figure 4:**
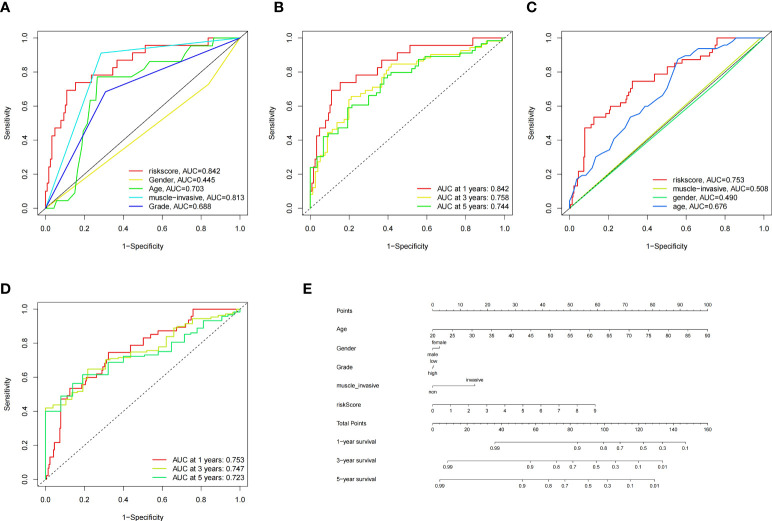
ROC Curves of the Model in GSE13507 and TCGA Datasets, and Nomogram. **(A)** Multiple-index ROC curves for GSE13507 patients, **(B)** Time-dependent ROC curve ROC curves for GSE13507 patients, **(C)** Multiple-index ROC curves for TCGA patients, **(D)** Time-dependent ROC curve ROC curves for TCGA patients, **(E)** Nomogram build based on training cohort.

To construct BCa related prognostic model, the univariate cox regression analysis was adopted to screen out prognostic genes. According to the P-value <= 0.01 standard, twenty-one prognostic related genes were screened (**Figure 2A**). Eight prognostic genes were further analyzed using Lasso regression (**Figures 2B, D**). Because Lasso regression only helped us compress the variables to 8 genes, but in the end, we used multivariate Cox regression to build a prognostic model that includes 4 genes: SERPINE2, SNCAIP, NMU, and S100A9. (**Supplementary Table 6**) (**Figure 2C**). Then we evaluated whether our model was an independent factor affecting patient prognosis. Because whether there was muscular infiltration was based on TNM staging, we did not include TNM staging in the evaluation, and since the model was based on whether there was muscular invasion, we compared whether there was muscular invasion and model genes to observe whether they were independent prognostic factors. Therefore, we performed univariate and multivariate independent prognostic analyses on age, sex, pathological grade, and whether there was muscular invasion, and finally found that our model was an independent factor affecting patient prognosis (P<0.01) (**Figures 2E, F**). Then, the risk score was calculated by formula (Specific values were included in **Supplementary Table 2**). The distribution of risk score, survival status, gene expression and the KM survival curve in the training set (**Figures 3A-D**) and testing set (**Figures 3E-H**) demonstrated a positive association between risk score and mortality. In addition, the AUC of the ROC curve was 0.842 in training cohort (GSE13507) (**Figure 4A**). Compared with other factors, the AUC of the model was the highest, indicating that our model had the best predictive ability for patient survival. The ROC curves for riskscore at 1, 2, and 3 years in the training dataset were 0.842, 0.758, and 0.744, respectively (**Figure 4B**). TCGA-BLCA dataset was adopted as an external validation dataset to validate the accuracy of model (**Supplementary Table 7**). We found that the diagnostic ability of the model was significantly higher than that of other factors (**Figure 4C**). The AUC of the 1, 2, and 3-year OS of riskscore in the TCGA-BLCA dataset was 0.753, 0.747, and 0.723, respectively (**Figure 4D**). At the same time, multiple-index ROC analysis showed that age, sex, tumor grade, whether there was muscular infiltration, and risk score may all have an impact on patient prognosis, so we also constructed a nomogram based on age, sex, whether there was muscular invasion, tumor pathology grade, and risk score to predict the patient’s 1-year, 3-year, and 5-year survival rates (**Figure 4E**).

### 3.4 Single-cell sequencing data and microarray data validate the differential gene expression of the model

**Figure 5:**
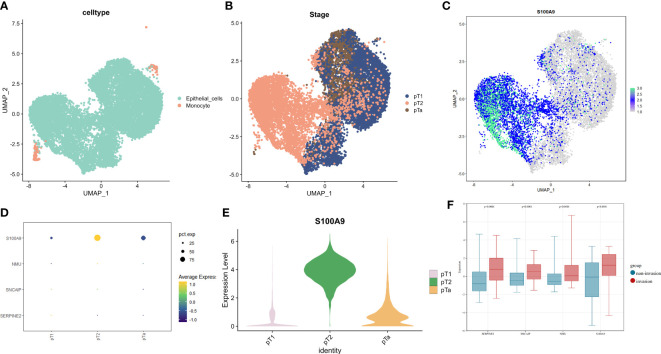
The expression profiles of model genes in single-cell data and GSE32894 dataset(revised). **(A)** UMAP dimensionality reduction displayed by cell type, **(B)** UMAP dimensionality reduction displayed by sample stage in epithelial cells. **(C)** Expression of S100A9 on UMAP dimensionality reduction in epithelial cells. **(D)** Expression levels of model genes in single-cell data based on sample T stage. **(E)** Violin plots showing the expression of S100A9 on UMAP in samples of pT1, pT2, and pTa stages. **(F)** Expression of model genes involved in muscle-invasive and non-muscle invasive bladder cancer in GSE32894.

To validate the expression of genes in our model in muscle-invasive and non-muscle-invasive bladder cancer, we utilized single-cell sequencing data. Firstly, we downloaded the expression matrix of the single-cell data, including pTa (GSM4006644), pT2 (GSM4006647), and pT1 (GSM4006645), where pT1 and pTa represent non-muscle-invasive BCa, and pT2 represents muscle-invasive BCa. Subsequently, we performed data analysis using the Seurat package in R programming language. We defined the number of highly variable genes as 3000. Finally, we conducted visualization-based dimensionality reduction using the obtained highly variable genes and principal components (PCs). For this purpose, we employed the UMAP method as our dimensionality reduction technique (**Figure 5A**). We integrated the data using the FindIntegrationAnchors function and identified two cell types: epithelial cells and monocytes. Since BCa primarily originates from epithelial cells, we extracted the epithelial cells separately and displayed their distribution in each BCa type (**Figure 5B**). We examined the expression of model-associated genes in pTa, pT2, and pT1 to investigate the expression differences of model genes between muscle-invasive and non-muscle-invasive BCa. We observed significant expression differences in S100A9, with higher levels observed in patients with muscle-invasive BCa (**Figures 5C, E**). However, due to the limited sequencing depth in the single-cell data, the detection rates of SERPINE2, SNCAIP, and NMU were relatively low (**Figure 5D**). As the expression data for non-muscle-invasive BCa in TCGA was limited, we further validated our results using a microarray dataset (GSE32894, **Supplementary Table 8**). First, we downloaded the gene expression matrix and grouped patients based on T-stage (**Supplementary Table 4**). The data had already been log2-transformed and standardized, resulting in gene expression values with negative values. By extracting and comparing the expression of model-related genes, we found significant differences in gene expression between muscle-invasive and non-muscle-invasive BCa patients in the model (**Figure 5F**). Moreover, the expression of model genes was significantly higher in patients with muscle-invasive BCa, consistent with our previous differential analysis and single-cell sequencing analysis. In the model, the risk coefficients of the relevant genes were all positive, consistent with the increased expression trend of model genes in muscle-invasive BCa in the validation dataset. These findings indicate the reliability of our model construction. Combined with previous clinical data analysis, we have reason to believe that the identified model genes play an important role in predicting the risk of muscle invasion and prognosis in BCa patients.

### 3.5 Identification of immunoinfiltration features of the prognostic model

**Figure 6:**
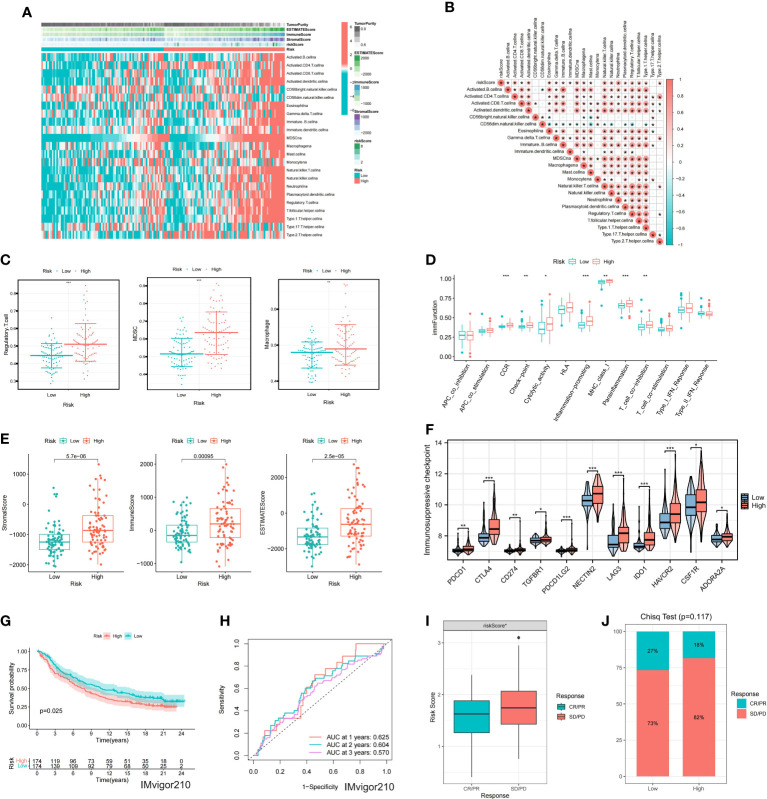
Immune characteristics of the model **(A)** Heatmap showing ssGSEA results for high and low-risk groups. **(B)** Correlation analysis between risk score and immune cells. **(C)** Differential expression of immunosuppressive cells between high and low-risk groups. **(D)** Differential expression of immune function scores within the model. **(E)** Differences in TME scores between high and low-risk groups. **(F)** Differential expression of immunosuppressive checkpoints between high and low-risk groups. **(G)** KM survival analysis for the model in the IMvigor210. **(H)** ROC curves for the model at 1, 2, and 3 years. **(I)** Differential expression of riskscore between CR/PR and SD/PD groups. **(J)** Differences in the proportion of CR/PR and SD/PD between high and low-risk groups. * represents p< 0.05; ** represents p< 0.01; *** represents p< 0.001.

As BCa is known to be an immunoresponsive tumor with high heterogeneity and metastatic potential, we further investigated the prognostic model of the immune microenvironment characteristics. The heatmap showed the distribution of the TME scores and immune cells between high and low risk groups (**Figure 6A**). The association between the immune infiltration cells and riskscore was illustrated in **Figure 6B**, revealing a strong relationship between riskscore and immune cells. Furthermore, the expression levels of immunosuppressive cells, including Myeloid-derived suppressor cells (MDSCs), Regulatory T cells, and macrophages, were found to be significantly higher in the high risk group (**Figure 6C**). Moreover, the scores of immune-related molecules such as Checkpoint, CCR, and Inflammation-promoting molecules were significantly elevated in the high risk group compared to the low risk group (**Figure 6D**). Additionally, we analyzed the potential relationship between riskscore and tumor microenvironment scores. The ESTIMATE Score, Stromal Score, and Immune Score were significantly higher in the high risk group (**Figure 6E**). We used the reshape2 package in R to analyze and compare the immunosuppressive checkpoints of high- and low-risk groups. Most of the differentially expressed immunosuppressive checkpoints have higher expression levels in the high-risk group than in the low-risk group (**Figure 6F**).

### 3.6 Immunotherapy and drug sensitivity analysis of the prognostic model

**Figure 7:**
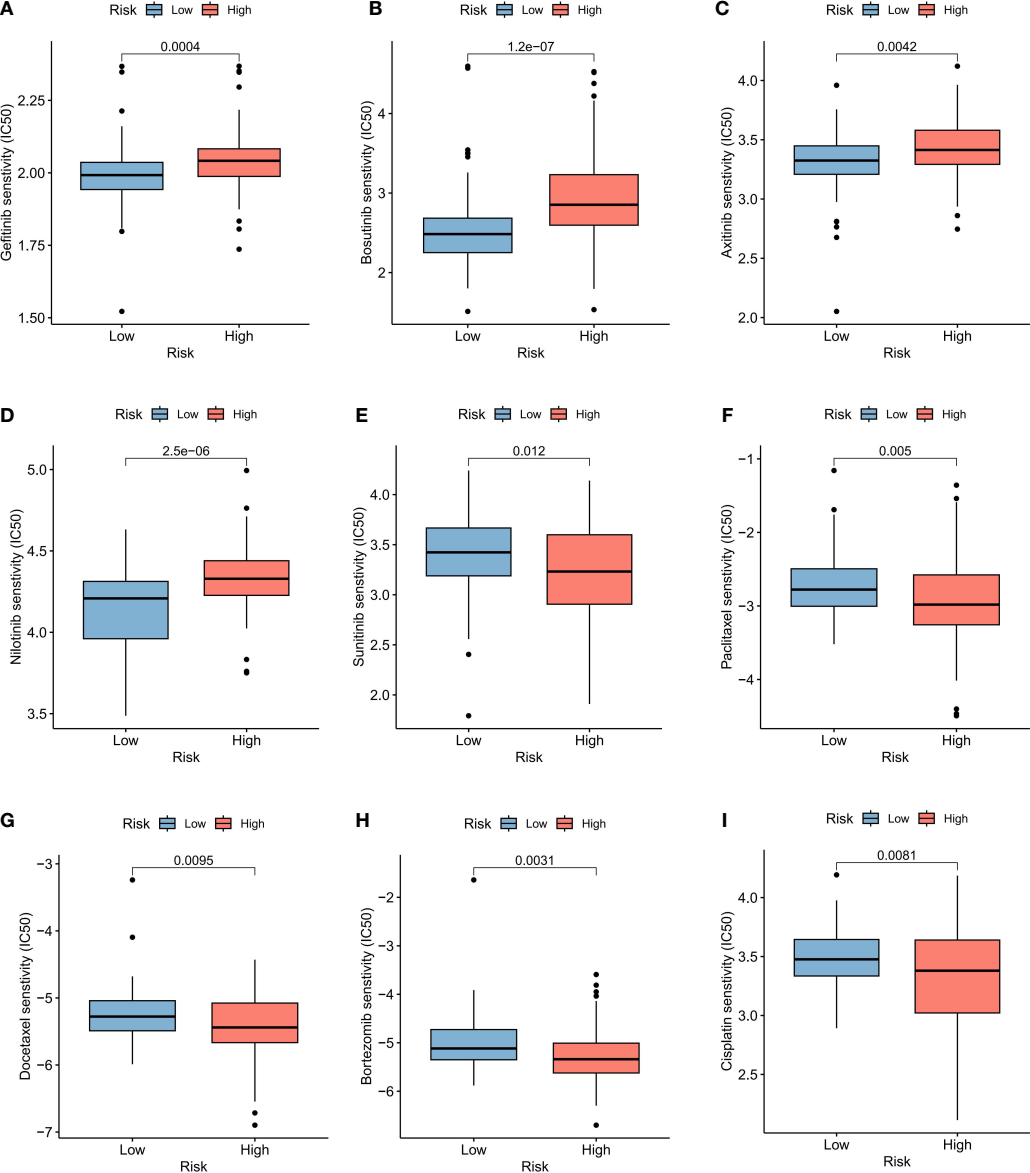
Drug sensitivity of BCa prognostic model. Sensitivity analysis for Gefitinib **(A)**, Bosutinib **(B)**, Axitinib **(C)**, Nilotinib **(D)**, Sunitinib **(E)**, Paclitaxel **(F)**, Docetaxel **(G)**, Bortezomib **(H)** and Cisplatin **(I)** between low and high risk groups.

In order to evaluate the model’s response to immunotherapy, we validated the association of risk scores with immunotherapy in the BCa immunotherapy dataset (IMvigor210 cohort). As a result, we found poor prognosis in the high-risk group in the immunotherapy dataset (IMvigor 210) (**Figure 6G**). The AUC of the 1, 2, and 3-year OS of riskscore in the IMvigor 210 dataset was 0.625, 0.604, and 0.57, respectively (**Figure 6H**). **Figure 6I** indicated that the proportion of SD and PD in the high risk group was higher. Meanwhile, riskscore was significantly over-expressed in the SD/PD group (**Figure 6J**). To further guide the development of clinical treatment strategies, we screened 9 major chemotherapeutic agents from the pRRophetic package to determine whether riskscore was associated with BCa resistance. The IC50 of Gefitinib, Bosutinib, Axitinib and Nilotinib was higher in high risk group, suggesting that these 4 drugs may be more suitable for patients with lower riskscore (**Figures 7A-D**). The IC50 of Sunitinib, Paclitaxel, Docetaxel, Bortezomib and Cisplatin was higher in low risk group (**Figures 7E-I**). These findings not only provide valuable insights for the selection of appropriate chemotherapy drugs according to the risk score of BCa patients, but also help to make clinical treatment decisions.

### 3.7 Overexpression of S100A9 in muscle-invasive and non-muscle-invasive BCa tissues

**Figure 8:**
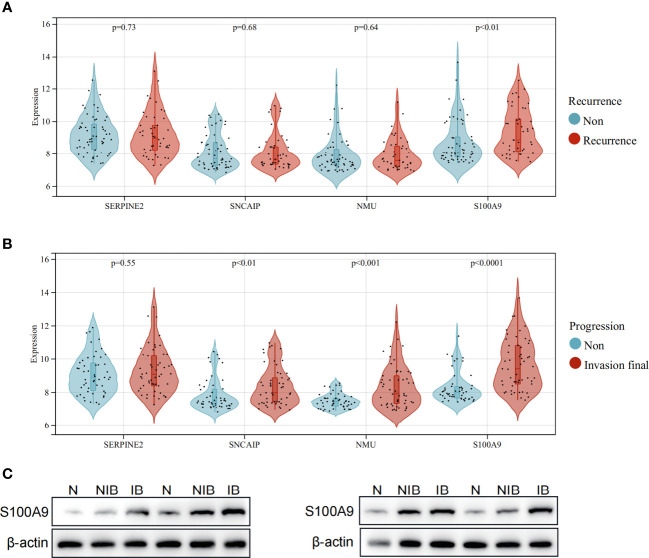
Expression of model genes in recurrent and progressive patients, and verification of protein expression levels of S100A9 in BCa. **(A)** Expression comparison of model genes between patients with no recurrence and recurrent patients. **(B)** Expression comparison of model genes between patients who did not progress to muscle-invasive BCa and patients who progressed to muscle-invasive BCa. **(C)** Western blot analysis showing the difference of S100A9 protein expression among normal, non-muscle-invasive and muscle-invasive BCa tissues.

In the cohort used to construct the model (GSE13507), clinical data on the subsequent progression of BCa patients were available. We found that the expression of S100A9 was significantly elevated in recurrent patients (**Figure 8A**). Furthermore, in patients who progressed to muscle-invasive BCa compared to those who did not, the expression of S100A9 was also significantly increased (**Figure 8B**). Additionally, single-cell sequencing data demonstrated higher expression of S100A9 in epithelial cells of muscle-invasive BCa compared to non-muscle-invasive BCa. Therefore, we selected S100A9 from the model as the target for further experimental validation. To validate the biological function of S100A9 in BCa, we first evaluated the protein expression of S100A9 among normal, muscle-invasive and non-invasive BCa tissues. WB showed that the protein expression of S100A9 was higher in muscle-invasive and non-invasive BCa than in normal tissues, and the expression was the highest in muscle-invasive BCa (**Figure 8C**).

### 3.8 S100A9 promotes BCa cell proliferation, migration and invasion *in vitro*

**Figure 9:**
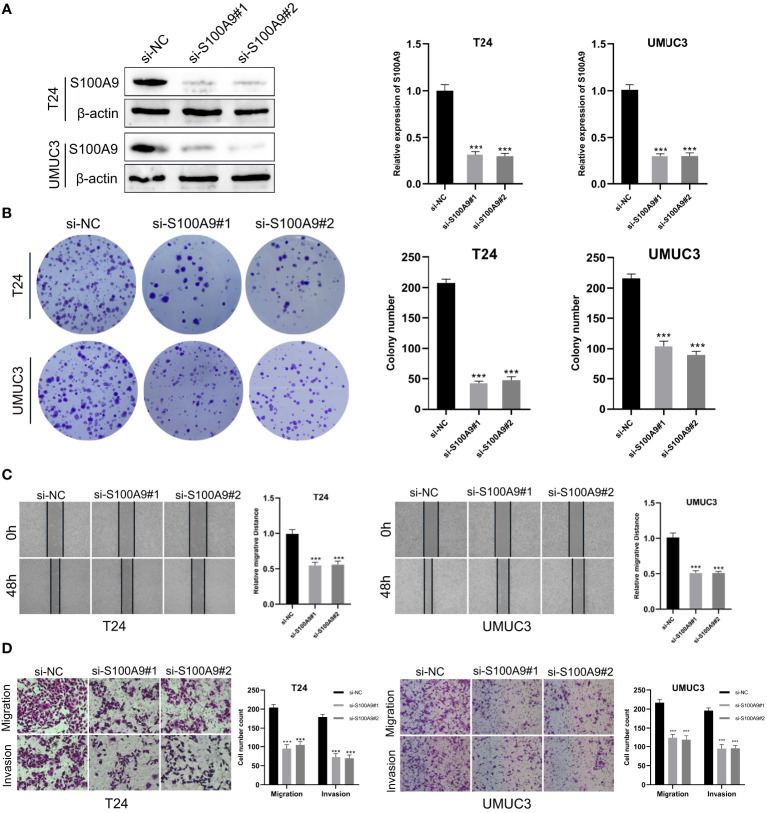
Downregulation of S100A9 suppressed the progression of BCa *in vitro*. **(A)** The protein expression of S100A9 was downregulated in T24 and UMUC3 cells, as determined by Western blot; **(B)** Colony formation assay in si-S100A9 and control cells; **(C)** Wound healing assay was used to detect the effect of S100A9-knockdown on BCa cell migration. Cell migration ability was represented by the wound gap distance in microscopic field at the time points of 0 and 48 h; **(D)** S100A9-knockdown suppressed BCa cell metastasis in T24 and UMUC3. *** represents p<0.001.

First, S100A9-siRNA was transfected into T24 and UMUC3 cells to knock down S100A9. The results indicated that the si-S100A9 used in the experiment effectively suppressed the expression of S100A9 (**Figure 9A**). Colony formation experiments confirmed that si-S100A9 significantly impaired the proliferative capacity of T24 and UMUC3 cells (**Figure 9B**). The transwell assay demonstrated that si-S100A9-treated T24 and UMUC3 cells exhibited reduced migration and invasion capacities compared to the control group (**Figures 9C, D**). This was consistent with the results of our previous analysis, indicating that S100A9 played a role in promoting proliferation, migration and invasion in BCa cells.

## 4 Discussion

With the rapid development of genomic technologies, the cost of sequencing is also decreasing. The cost of single-sample sequencing is now under $100,especially for clinical exome sequencing (CES), and if a unified platform is used for sequencing, the cost is likely to decrease further (11). This is a small fraction of the long-term follow-up and treatment costs for BCa patients. In addition, standardized sequencing at scale will standardize the various gene expression values that affect patient prognosis. In previous studies, although there have been differential analysis and gene identification of muscle-invasive and non-muscle-invasive bladder cancer based on databases, all of their research was solely database-driven. While seven genes, including S100A9, were investigated and validated in databases, no experimental verification was conducted. The identified genes were not further validated in the normal, non- muscle-invasive bladder cancer, and muscle-invasive bladder cancer groups. Our research addressed these shortcomings by conducting experimental validation and utilizing single-cell sequencing data for further verification. Additionally, we explored the impact of the S100A9 gene on bladder cancer cells in cell lines, thereby advancing research in this field. In our study, because the sequencing platforms and expression data processing methods are different, there may be differences in the numerical expressions. Therefore, when studying the impact of risk scoring on patients’ overall survival rates, we used median values to group high- and low-risk patients from both GSE13507 and TCGA patients. And we build nomogram only based on training cohort (GSE13507). Additionally, as the use of cystoscopy is essential for the diagnosis and treatment of BCa, obtaining sequencing samples is also extremely simple. Assuming that the standardized sequencing platform is successfully established, reference values for the standardized expression of various genes are available, which provides more options for the treatment and follow-up of BCa patients. For example, early-stage BCa patients can take aggressive treatment based on the high expression of certain invasive genes, thereby avoiding the risk of muscle infiltration. This approach can reduce patients’ treatment costs and improve treatment efficacy. For patients with invasive BCa, based on the existing bioinformatics algorithms, their immune checkpoint status and tumor microenvironment can be estimated according to their gene expression information, the degree of infiltration can be observed, and personalized immunotherapy can be carried out accordingly. In recent years, there have been significant advances in research on immune therapy and targeted therapy for BCa (27). More accurate sequencing methods such as single cell sequencing and spatial transcriptome have also been added to the search for BCa markers (28). Precise sequencing can target specific cancer cell molecules or immune cells for targeted therapy, thereby improving treatment efficacy and prognosis.

In this study, we focused on using differential genes between muscle-invasive and non-muscle-invasive BCa to develop and validate prognostic features of BCa. First, 183 MIRDGs were identified between muscle-invasive and non-muscle-invasive BCa. Secondly, based on multivariate cox regression analysis, four genes (SERPINE2, SNCAIP, NMU, S100A9) were determined as prognostic features. At the same time, the KM survival curve in the model also showed that the survival time of the low-risk group of patients was significantly better than that of the high-risk group. The survival results were independently validated using the TCGA dataset. and the AUC of the ROC curve was also good, and the survival curve results were consistent. Furthermore, single-cell sequencing data further confirmed that the S100A9 in the model had differential expression between the two types of cancer, and S100A9 in invasive BCa was significantly higher than that in non-invasive BCa. Because the depth of single-cell sequencing was insufficient, we used microarray data (GSE32894) for further validation. The results showed that the expression of the model genes was higher in the muscle-invasive BCa group than in the non-muscle-invasive bladder group, indicating that our model genes were consistent with the differential analysis we previously performed using GSE13507. Moreover, this is consistent with the calculation we obtained when building the model, where HR>1, indicating that high expression may lead to poor prognosis of BCa. In addition, immunosuppressive checkpoints and immunosuppressive cells were significantly overexpressed in the high-risk group. At the same time, there were significant differences in StromalScore, ImmuneScore, ESTIMATEScore, TumorPurity between the high- and low-risk groups. These results suggested that four model genes may be involved in constructing immunosuppressive microenvironments that promote tumor invasion and metastasis. The upregulation of immune checkpoints in the high-risk group suggests a stronger ability to evade immune surveillance, which may contribute to the poor prognosis in this group. Notably, we observed that the expression of PD-1-related immune checkpoints (CD274, PDCD1) showed significant differences but overall low expression levels. This finding is consistent with previous studies that have reported BCa’s insensitivity to PD-1 therapy, further confirming the accuracy of our analysis. Furthermore, the higher immune scores in the high-risk group compared to the low-risk group indicate significant differences in the tumor microenvironment between these groups. This disparity may be an important factor contributing to the unfavorable prognosis observed in the high-risk group.

Our prognostic signature include four genes, SERPINE2, SNCAIP, NMU, and S100A9, each playing a critical role in tumor progression, invasion, and metastasis. SERPINE2 is a member of the serine protease inhibitor family and is mainly expressed in the placenta, brain, and urothelial epithelium. Upregulation of SERPINE2 has been reported to increase the radioresistance of lung cancer cells and is also involved in the invasion and metastasis of endometrial cancer (29). High expression of SERPINE2 indicates poor prognosis of urothelial carcinoma, which is consistent with the results of our study (30), and it also promotes tumorigenesis in various cancers (31, 32). SNCAIP encodes synaptic nuclear protein α-interacting protein and has been found to be highly expressed in metastatic clear cell carcinoma (33). The NMU encodes a member of the neuropeptide neuromedin family. The encoded protein is a precursor that is processed by proteolytic cleavage to produce biologically active neuropeptides that play a role in pain, stress, and immune-mediated inflammatory diseases (34). Increased expression of this gene has been observed in kidney cancer (35), pancreatic cancer (36), and lung cancer (37). S100A9 has been found to be a protein that bridges inflammation and cancer. Increased expression of S100A9 is considered a sign of increased tumor proliferation and invasive ability and is believed to be a new therapeutic target for cancer treatment (38).

The occurrence of recurrence and progression in BCa treatment is a major concern for clinicians. Recurrence not only necessitates additional surgical interventions but also requires close monitoring and follow-up, which can impose a significant burden on patients in terms of time and financial resources. Progression to muscle-invasive disease leads to a worse prognosis, requiring more aggressive and invasive treatments such as bladder removal surgery, which rapidly diminishes the patient’s quality of life. In our analysis of the GSE13507 cohort, we observed elevated expression of S100A9 in both recurrent and progressive BCa cases. Furthermore, single-cell data revealed increased expression of S100A9 in invasive BCa within the extracted epithelial cells. We conducted further experimental validation, including western blot, which confirmed high protein expression of S100A9 in invasive BCa patients. Cell-based assays demonstrated that silencing S100A9 reduced the invasiveness of BCa cells. These findings suggest that S100A9 may serve as a prognostic marker for early-stage BCa patients.

In non-invasive BCa patients with high expression of S100A9, more aggressive and timely treatments such as partial bladder resection or frequent follow-up should be considered. However, this decision requires validation through large-scale clinical trials. In other studies, increased expression of S100A9 has been observed in urine samples from both invasive and non-invasive BCa patients, accompanied by cell-based experiments. Our study, based on clinical information from patients, not only identified S100A9 but also provided an explanation for its elevated expression in invasive BCa and its association with recurrence and progression. This suggests that the diagnostic use of S100A9 during the initial assessment of bladder lesions would greatly assist clinicians in predicting patient prognosis. This is the innovative aspect of our research. However, our current study has certain limitations. Firstly, the data collected were from public databases, and the sample size was limited. Future research should overcome these limitations by employing larger sample sizes. Additionally, while we validated the performance of S100A9 in cell experiments and clinical samples, the specific mechanisms and the roles of the other three genes in our model remain unknown. Future studies will follow the following plans: designing more comprehensive clinical trials with well-defined clinical endpoints, such as the number and timing of recurrences in non-muscle-invasive BCa and the conversion to muscle-invasive disease, and their respective time points. Moreover, a standardized sequencing workflow will be designed to collate sequencing data for unified comparative analysis, which can be matched with newly added clinical events to more accurately identify genes influencing patient prognosis and provide treatment targets. Finally, the identified genes possess complex functionalities and molecular mechanisms, which will require further validation in cellular and animal models.

## 5 Conclusion

In this study, we further elucidated the role of differential genes in prognosis between muscle-invasive and non-muscle-invasive BCa. Moreover, we have constructed the prognostic model in BCa patients, which may be employed as a reliable predictor of prognosis and immune response.

Meanwhile, S100A9 may promote the proliferation, migration and invasion of BCa cells, which may be a potential therapeutic target of BCa.

## Data availability statement

The datasets presented in this study can be found in online repositories. The names of the repository/repositories and accession number(s) can be found within the article/**Supplementary Material**.

## Ethics statement

All the patients provided written informed consent, and the protocol was approved by ethical committee of Suzhou Municipal Hospital. The studies were conducted in accordance with the local legislation and institutional requirements. The participants provided their written informed consent to participate in this study.

## Author contributions

KZ and WW designed this work. XZ and SJ wrote the manuscript. WW performed the bioinformatics analysis. PX performed the data review. KC, KL, FW, and XL provided administrative and financial support. All authors contributed to the article and approved the submitted version.
